# Comparative study between colonic metallic stent and anal tube decompression for Japanese patients with left-sided malignant large bowel obstruction

**DOI:** 10.1186/s12957-018-1509-0

**Published:** 2018-10-17

**Authors:** Satoru Kagami, Kimihiko Funahashi, Mitsunori Ushigome, Junichi Koike, Tomoaki Kaneko, Takamaru Koda, Akiharu Kurihara, Yasuo Nagashima, Yu Yoshino, Mayu Goto, Tetsuo Mikami, Kumiko Chino

**Affiliations:** 1 Department of General and Gastroenterological Surgery, Toho University Omori Medical Center, 6-11-1 Omorinishi Otaku, Tokyo, 143-8541 Japan; 2 Department of Pathology, Toho University School of Medicine, 5-21-16 Omorinishi, Otaku, Tokyo, 143-8540 Japan; 3 Department of Internal Medicine, United States Naval Hospital, 1-chome Tomari-cho, Yokosuka-shi, Kanagawa, 238-0001 Japan

**Keywords:** Left-sided malignant large bowel obstruction, Colonic metallic stent, Trans-anal decompression tube, Short- and mid-term outcomes

## Abstract

**Background:**

Surgical management of malignant bowel obstruction carries with high morbidity and mortality. Placement of a trans-anal decompression tube (TDT) has traditionally been used for malignant bowel obstruction as a bridge to surgery. Recently, colonic metallic stent (CMS) as a bridge to surgery for malignant bowel obstruction, particularly left-sided malignant large bowel obstruction (LMLBO) caused by colorectal cancer, has been reported to be both a safe and feasible option. The aim of this retrospective study is to evaluate the clinical effects of CMS for LMLBO as a bridge to surgery compared to TDT.

**Methods:**

Between January 2000 and December 2015, we retrospectively evaluated outcomes of 59 patients with LMLBO. We compared the outcomes of 26 patients with CMS for LMLBO between 2013 and 2015 (CMS group) with those of 33 patients managed with TDT between 2003 and 2011 (TDT group) by the historical study. LMLBO was defined as a large bowel obstruction due to a colorectal cancer that was diagnosed by computed tomography and required emergent decompression.

**Results:**

All patients in the CMS group were successfully decompressed (*p* = 0.03) and could initiate oral intake after the procedure (*p* <  0.01). Outcomes in the CMS group were superior to the TDT group in the following areas: duration of tube placement (*p* <  0.01), surgical approach (*p* <  0.01), operation time (*p* <  0.01), number of resected lymph nodes (*p* <  0.001), and rate of curative resection (*p* <  0.01). However, no significant differences were found in the overall postoperative complication rate (*p* = 0.151), surgical site infection rate (*p* = 0.685), hospital length of stay (*p* = 0.502), and the need for permanent ostomy (*p* = 0.745). The 3-year overall survival rate of patients in the CMS and TDT groups was 73.0% and 80.9%, respectively, and this was not significant (*p* = 0.423).

**Conclusions:**

Treatment with CMS for patients with LMLBO as a bridge to surgery is safe and demonstrated higher rates of resumption of solid food intake and temporary discharge prior to elective surgery compared to TDT. Oncological outcomes during mid-term were equivalent.

## Background

Malignant large bowel obstruction is caused by a variety of advanced malignancies, and has traditionally been approached surgically with colonic resection and possible stoma creation or large intestine bypass. However, surgical management of malignant large bowel obstruction carries with high morbidity and mortality, high stoma creation rate, and prolonged hospital stay. Recently, colonic metallic stent (CMS) as a bridge to surgery and also for palliation for malignant large bowel obstruction, particularly left-sided malignant large bowel obstruction (LMLBO) caused by colorectal cancer, has been reported to be both a safe and feasible option [1]. However, CMS is not recommended routinely by the European Society of Gastrointestinal Endoscopy guidelines because of its lack of safety [2]. In Japan, placement of a trans-anal decompression tube (TDT) has traditionally been used for LMLBO as a bridge to surgery. Since CMS for malignant large bowel obstruction has been covered by insurance since 2012 in Japan, its feasibility has recently been evaluated. However, the clinical efficacy of CMS for LMLBO is not clear as there are few reports of comparative outcomes between CMS and TDT for LMLBO [3, 4].

The aim of this retrospective study is to evaluate the surgical outcomes and short- and mid-term results of CMS for Japanese patients with LMLBO compared with TDT.

## Methods

We retrospectively evaluated surgical outcomes and short- and mid-term results of 26 patients with CMS for LMLBO as a bridge to surgery (CMS group, treated between 2013 and 2015) and compared with those of 33 patients with TDT (control group, treated between 2003 and 2011). In this study, LMLBO was defined as a large bowel obstruction caused by a colorectal cancer that was diagnosed by computed tomography in which urgent decompression was deemed necessary.

Regarding TDT insertion, the ArgyleTM Denis Colorectal tube (Medtronic Corp.) was used. We forcibly irrigated the large bowel using water twice daily for approximately 1 week until a planned surgery. Regarding CMS placement, we treated LMLBO with either the WallFlexTM Colonic Stent 22 mm (Boston Scientific Corp.) or the Niti-STM 22 mm (TaeWoong Corp.). Both TDT insertion and CMS placement were performed by colorectal surgeons. The insertion technique was via a combined endoscopic and fluoroscopic approach.

Postoperative complications were defined according to the Clavien-Dindo classification system. A hospital stay was defined as a duration to discharge after surgery in this study.

### Statistical analysis

Quantitative data are reported as median (range). The Mann-Whitney *U* test was used to compare continuous variables, and chi-square or Fisher’s exact tests were used to compare discrete variables. *P* values less than 0.05 were considered statistically significant.

## Results

### Patient characteristics

Patient characteristics are shown in Table 1. A total of 59 patients including 40 males were evaluated retrospectively. Median age was 69 years (range, 46–90 years). Most chief complaint was abdominal pain and/or abdominal fullness, which is similar between the two groups. Median maximum of dilatation of the colon of the TDT group and the CMS group were 59.9 mm (range, 33.0–95.3 mm) and 48.8 mm (range, 29.2–76.8 mm), respectively. Dilatation of the small bowel was found in 16 patients in the TDT group and in 11 patients in the CMS group, respectively. No statistical differences were found between the two groups. Twenty-six (44.1%) of 59 patients with LMLBO were treated with CMS. Clinicopathologically, 38 (64.4%) patients had advanced cancer, including 20 patients with distant metastases.

**Table 1:** Patient characteristics

	Total (*n* = 59)	TDT (*n* = 33)	CMS (*n* = 26)	*p* value
Gender				0.939
Male	40	23	17	
Female	19	10	9	
Age (range)*	69 (46–90)	68 (46–90)	70 (50–85)	0.367
Diabetes mellitus				0.180
Positive	15	11	4	
Negative	44	22	22	
Body mass index, kg/m^2^ (range)*	–	20.9 (17.0–41.5)	20.7 (13.2–29.3)	0.803
Chief complaint				–
Abdominal pain/fullness	34	21	13	
Vomiting	4	3	1	
Constipation	5	3	2	
Bloody stool	7	3	4	
Diarrhea	1	1	0	
Anemia	4	0	4	
Others	4	2	2	
Tumor location				0.366
Transverse	2	0	2	
Descending	9	6	3	
Sigmoid	38	21	17	
Rectosigmoid	10	6	4	
Dilatation of the small bowel				
Positive	27	16	11	0.749
Negative	32	17	15	
Maximum of dilatation of the colon, mm (range)*	–	59.9 (33.0–95.3)	48.8 (29.2–76.8)	0.099
CEA, ng/ml (range)*		10.0 (2.9–490.8)	6.6 (1.0–1232.0)	0.242
TDT	33	–	–	
CMS	26	–	–	
Stage				0.441
II	22	11	11	
III	17	11	6	
IV	20	11	9	

*median, TDT = trans-anal decompression tube, CMS = colonic metallic stent

### Clinical outcomes

Clinical outcomes are shown in Table 2. For all 26 patients who were treated with CMS, CMS was deployed without technical issue. Additionally, for all 26 patients undergoing CMS placement, resumption of a regular diet and temporary discharge were possible. On the other hand, 6 (18.2%) of 33 patients treated with TDT had clinical failure in the form of intestinal perforation, stent migration, or incomplete decompression (3 (9.1%) patients, 2 (6.1%) patients, and 1 (3.0%) patient, respectively). For the three patients with perforation, surgical exploration was performed immediately; for two of these patients, primary tumors were resected and stomata were created; for one patient, left hemicolectomy was performed without stoma creation. Technically successful tube deployment was achieved in 27 (81.8%) of 33 patients with TDT. The duration after initial decompression to surgery in the CMS and TDT groups was 17 days (range, 6–54 days) and 9 days (range, 1–30 days), respectively. This difference was statistically significant (*p* <  0.01).

**Table 2:** Clinical outcomes

	TDT group (*n* = 33)	CMS group (*n* = 26)	*p* value
Clinical success (%)	27 (81.8)	26 (100)	0.03
Clinical failure (%)	6 (18.2)	0	
Perforation	3 (9.1)	0	
Migration	2 (6.1)	0	
Inadequate decompression	1 (3.0)	0	
Solid food intake			< 0.01
Resumed	0	26	
Not resumed	33	0	
Temporary discharge			< 0.01
Yes	0	26	
No	33	0	
Duration of tube placement, days (range)*	17 (6–54)	9 (1–30)	< 0.01

*median, TDT = trans-anal decompression tube, CMS = colonic metallic stent

Surgical outcomes are shown in Table 3. Surgery was performed laparoscopically for 20 (76.9%) patients in the CMS group (*p* <  0.01). For all patients who were treated with TDT, open surgery was chosen because inadequate colonic lavage was suspected preoperatively. Median operative time in the CMS group was significantly longer than that in the TDT group (367 min vs. 205 min; *p* <  0.01). Postoperative complications higher than grade 2 according to the Clavien-Dindo classification system occurred in five (15.1%) patients in the TDT group and nine (34.6%) patients in the CMS group (*p* = 0.151). Surgical site infection occurred in four (12.1%) patients in the TDT group and two (7.7%) patients in the CMS group (*p* = 0.685). Median overall hospital stay in the TDT group was similar to that in the CMS group (28 days vs. 27.5 days, *p* = 0.502). Regarding stoma creation during the primary operation, stoma was created for 12 (36.4%) patients in the TDT group and for 8 (30.8%) patients in the CMS group. Two patients in the CMS group and two patients in the TDT group eventually went on to stoma reversal. There were no significant differences in the rate of permanent stoma creation between the TDT group and the CMS group (30.3% vs. 23.1%, respectively; *p* = 0.745).

**Table 3:** Surgical outcomes

	TDT group (*n* = 33)	CMS group (*n* = 26)	*p* value
Surgical approach			< 0.01
Laparoscopic (%)	0	20 (76.9)	
Open (%)	33 (100)	6 (23.1)	
Surgical procedure			
Partial resection	1	0	–
Left hemicolectomy	4	3	
Sigmoidectomy	8	1	
Hartmann procedure	7	6	
Anterior resection	13	16	
Operative time, minutes (range)*	205 (100–447)	367 (210–597)	< 0.01
Blood loss, g (range)*	205 (0–1275)	102 (0–1492)	0.369
Stoma creation during primary operation (%)	12 (36.4)	8 (30.8)	0.862
Postoperative complications (%) (the Clavien-Dindo classification)			0.151
Grade 0	24 (72.7)	16 (61.5)	
1	4 (12.1)	1 (3.9)	
2	4 (12.1)	7 (26.9)	
3	1 (3.1)	2 (7.7)	
4	0 (0)	0 (0)	
Surgical site infection (%)			0.685
Positive	4 (12.1)	2 (7.7)	
Negative	29 (78.9)	24 (92.3)	
Hospital stay, days (range)*	28 (14–75)	27.5 (12–114)	0.502
Stoma reversal (%)	2 (6.1)	2 (7.7)	
Permanent stoma creation (%)	10 (30.3)	6 (23.1)	0.745

*median, TDT = trans-anal decompression tube, CMS = colonic metallic stent

### Clinicopathological outcomes

Clinicopathological outcomes are shown in Table 4. Median number of resected lymph nodes in the CMS group was 19 (range, 6–40 nodes) compared with 9 (range, 1–23 nodes) in the TDT group. This was significant (*p* <  0.01). Regarding curative resection of the primary tumor, rate of curative resection in the CMS group was superior to that in the TDT group, and this was significant (*p* <  0.01).

**Table 4:** Clinicopathological outcomes

	TDT group (*n* = 33)	CMS group (*n* = 26)	*p* value
Tumor size, cm (range)*	4.5 (3.0–9.0)	5.5 (3.5–11.0)	0.008
Histological type (%)			0.470
Tub 1	11 (33.3)	7 (26.9)	
Tub 2	20 (60.7)	16 (61.6)	
Por	1 (3.0)	1 (3.8)	
Muc	0 (0)	2 (7.7)	
Sci	1 (3.0)	0 (0)	
Stage (%)			0.441
II	11 (33.3)	11 (42.3)	
III	11 (33.3)	6 (23.1)	
IV	11 (33.3)	9 (34.6)	
Lymph nodes, n (range)*	9 (1–23)	19 (6–40)	< 0.01
Resection status (%)			< 0.01
D1	3 (9.1)	0 (0)	
D2	12 (36.4)	1 (3.8)	
D3	18 (54.5)	25 (96.2)	
Recurrence (%)			0.424
Yes	9 (40.9)	4 (23.5)	
No	13 (59.1)	13 (76.5)	
Observation period, days (range)*	1516 (17–4773)	608 (52–1601)	

*median, TDT = trans-anal decompression tube, CMS = colonic metallic stent

**Fig. 1:**
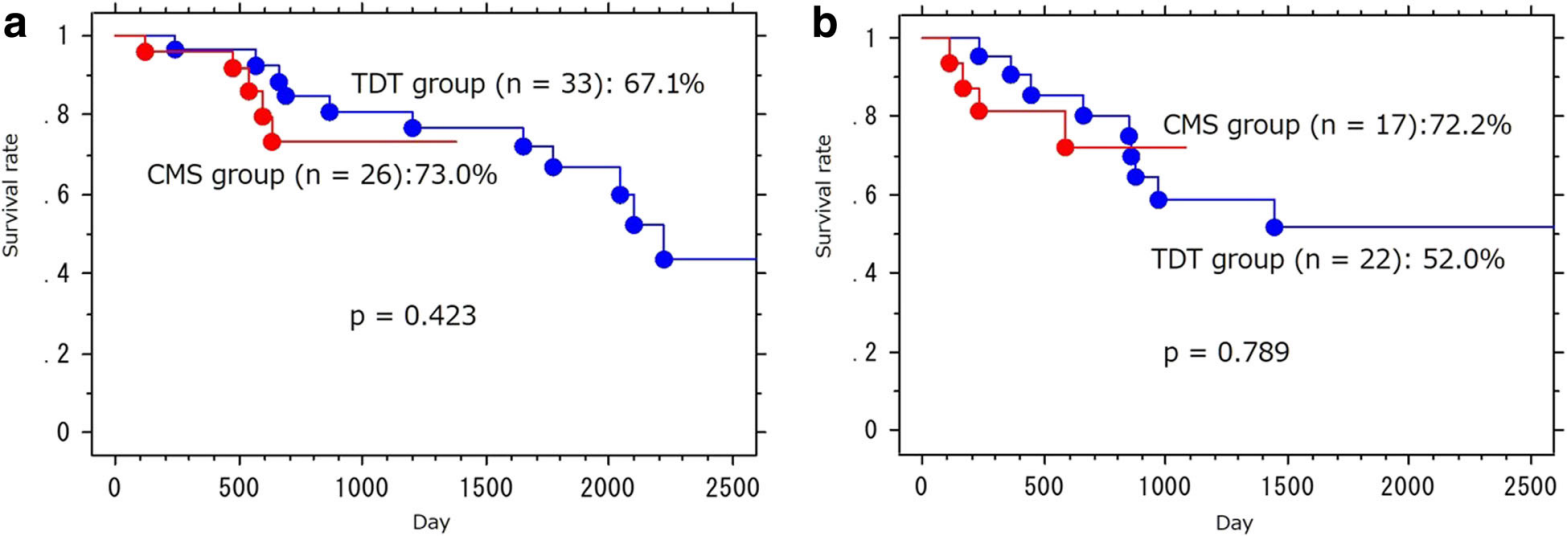
Survival curves of patients in the CMS group and the DTD group. Numeric values are showing 5-year overall survival rate (**a**) and 5-year disease-free survival rate in the two groups (**b**), respectively

The characteristics of adjuvant therapy are shown in Table 5. Regarding prognosis in patients with pathological stages of II and III, recurrence occurred in four (23.5%) patients in the CMS group and nine (40.9%) patients in the TDT group. The 5-year disease-free survival of pathological stage II and III patients in the CMS group and the TDT group was 72.2% and 52.0%, respectively (95% CI 2.43–2.98, *p* = 0.789). Furthermore, the 5-year overall survival rate of patients in the CMS group and the TDT group was 73.0% and 67.1%, respectively (95% CI 1.79–2.07, *p* = 0.423). This was not significant (Fig. 1).

**Table 5:** Characteristics of adjuvant therapy

	TDT group (*n* = 33)	CMS group (*n* = 26)
Adjuvant therapy		
Stage II		
Negative	7	6
Oral 5FU/leucovorin or oral 5FU	3	2
Capecitabine + oxaliplatin	0	3
mFOLFOX6	1	0
Stage III		
Negative	2	2
Oral 5FU/leucovorin or oral 5FU	7	1
Capecitabine + oxaliplatin ∓ bevacizumab	1	2
unknown	1	1
Stage IV		
Best supportive care	3	2
Oral 5FU/leucovorin or oral 5FU	1	2
mFOLFOX6 ∓ bevacizumab	3	2
mFOLFOX6 + panitumumab	0	1
Capecitabine + oxaliplatin ∓ bevacizumab	1	2
S1 + oxaliplatin + bevacizumab	1	0
Others	2	0

## Discussion

Malignant large bowel obstruction is caused by a variety of advanced malignant tumors, particularly pancreatic, gastric, colorectal, and peritoneal carcinomatosis with an ovarian primary. Malignant large bowel obstruction caused by colorectal cancer occurs in approximately 20% [5]: 9.0–17.8% in Japan. Traditionally, malignant large bowel obstruction caused by colorectal cancer has been approached surgically. For right-sided malignant large bowel obstruction, right hemicolectomy is performed, while for LMLBO, staged surgeries are usually necessary because the mortality for emergency surgery is much higher than that for elective surgery [6]. However, stoma that was created for initial decompression become permanent in patients with LMLBO because of the operative risk, advanced age, and patients’ unwillingness to proceed with further surgery. One-stage surgery with decompression including intraoperative lavage is thus an appealing option [7]. CMS and TDT strategies have been developed in order to achieve primary anastomosis without stoma at the initial surgery. Systematic reviews and meta-analyses have demonstrated the superiority of CMS for malignant large bowel obstruction as a bridge to surgery in terms of improved morbidity and shorter length of stay, among other benefits [8–11]. Currently, CMS is considered a feasible option for malignant large bowel obstruction as a bridge to surgery. As CMS for malignant large bowel obstruction has been covered by insurance since 2012 in Japan, its feasibility has been evaluated in the literature. Recently, two retrospective studies of comparative outcomes between CMT and TDT for LMLBO were reported in Japan, but the benefits were not clear. Kawachi et al. [3] reported that treatment with CMS had benefits in terms of decreased stoma creation rate, as well as high rates of technical and clinical success of the stenting procedure itself. However, significant differences were not found in terms of mortality, morbidity, and shorter hospital stay compared with TDT. Additionally, in the report by Matsuda et al. [2], there were no differences between the two groups in terms of stoma creation rate, mortality, and morbidity. The CMS group did, however, demonstrate higher QOL including shorter postoperative hospital stay, higher rates of solid food intake, and temporary discharge prior to surgery. On the other hand, Li et al. in China compared the TDT group (*n* = 13) with the CMS group (*n* = 16) for acute LMLBO to evaluate the clinical effects. They concluded that both TDT and CMS can achieve preoperative colonic lavage for 1-stage operation for patients with acute LMLBO with no increase in complications [12]. However, these results were retrospective, single-center, and were carried out with a relatively small group of patients.

Optimizing technical success and minimizing perforation are critical if applying CMS to patients with malignant large bowel obstruction. A meta-analysis by Allievi et al. [10] demonstrated that technical success rate and perforation rate using CMS were 78.8% and 5.9%, respectively. In this study, perforation was found only in the TDT group (*n* = 3, 9.1%), while a technical success rate of 100% was observed in the CMS group. Also, all patients treated with CMS reported higher rates of solid food intake and temporary discharge prior to surgery compared with patients treated with TDT. However, the occurrence of postoperative complications more than grade 2 and permanent stoma creation rate were equivalent. It was advantageous that all patients treated with CMS were able to initiate solid food intake and were able to be discharged from the hospital for a short time when compared to patients treated with TDT. Additionally, surgeries performed after CMS had more complete pathologic staging in terms of more resected lymph nodes. However, this study was a retrospective study, comparing the CMS group with the TDT group by the historical study. Therefore, there were differences in the background between two groups.

In the CMS group, there were no peritoneal recurrences that could be associated with technical failure or perforation during CMS insertion, and only one patient had a local recurrence after surgery. The 5-year overall survival rate, including the analysis of 9 patients with stage IV disease, and disease-free survival rate of pathological stage II and III patients in the CMS group were similar to those in the TDT group. Recently, the multicenter, randomized controlled ESCO trial showed there was no difference in oncologic outcomes with a median follow-up of 36 months [9]. Also, the meta-analysis by Matsuda et al. showed no significant difference between the CMS group and the emergency surgery group in terms of overall survival, disease-free survival, and recurrence. On the other hand, Sabbagh et al. [13] reported negative outcomes including ulceration near the tumor, perineural invasion, and lymph node invasion associated with CMS placement and that overall survival was significantly lower in the CMS group. Broholm et al. [14] reported that delay of surgery after stent placement for resectable malignant colorectal obstruction was associated with a higher risk of recurrence. Takahashi et al. reported that CMS placement increased plasma levels of cfDNA and ctDNA by tumor manipulation despite no management with TDT [15]. The oncologic consequence of CMS placement for MLBO remains unclear.

## Conclusions

This study indicates that treatment with CMS for LMLBO may have clinical benefits of its safety, higher rates of resumption of solid food intake, and temporary discharge prior to elective surgery compared to treatment with TDT. However, this study is limited by its small sample size, single-center retrospective design, and non-randomized nature. Furthermore, more than one type of stent was used. Future, randomized controlled trials are needed to clarify the superiority of treatment with CMS compared to TDT.

## Abbreviations

CMS: colonic metallic stent
MLBO: malignant large bowel obstruction
TDT: trans-anal decompression tube
